# IgM and IgG Profiles Reveal Peculiar Features of Humoral Immunity Response to SARS-CoV-2 Infection

**DOI:** 10.3390/ijerph18031318

**Published:** 2021-02-01

**Authors:** Antonella De Donno, Giambattista Lobreglio, Alessandra Panico, Tiziana Grassi, Francesco Bagordo, Maria Pia Bozzetti, Serafina Massari, Luisa Siculella, Fabrizio Damiano, Francesco Guerra, Marilena Greco, Michele Chicone, Roberta Lazzari, Pietro Alifano

**Affiliations:** 1 Department of Biological and Environmental Sciences and Technologies, University of Salento, 73100 Lecce, Italy; antonella.dedonno@unisalento.it (A.D.D.); alessandra.panico@unisalento.it (A.P.); francesco.bagordo@unisalento.it (F.B.); maria.bozzetti@unisalento.it (M.P.B.); sara.massari@unisalento.it (S.M.); luisa.siculella@unisalento.it (L.S.); fabrizio.damiano@unisalento.it (F.D.); pietro.alifano@unisalento.it (P.A.); 2 Clinical Pathology and Microbiology Unit, Vito Fazzi General Hospital, 73100 Lecce, Italy; gblobreglio59@gmail.com (G.L.); cescowar@gmail.com (F.G.); grecomarilena@gmail.com (M.G.); michelechicone@hotmail.it (M.C.); robertaa.lazzari@alice.it (R.L.)

**Keywords:** antibodies, COVID-19, humoral immunity, SARS-CoV-2

## Abstract

The emergence of coronavirus disease 2019 (COVID-19) is globally a major healthcare threat. There is little information regarding the mechanisms and roles of the humoral response in SARS-CoV-2 infection. The aim of this study was to analyze the antibody levels (IgM and IgG) by chemiluminescence immunoassay in 54 subjects positive to SARS-CoV-2 swab test in relation to their clinical status (whether asymptomatic, pauci-symptomatic or with mild, sever or critical symptoms), the time from the symptom onset, sex, age, and comorbidities. Overall, the presence of comorbidities and the age of subjects were associated with their clinical status. The IgG concentrations were significantly higher in patients who developed critical and severe symptoms and seemed to be independent from age, sex and comorbidities. IgG titers peaked around day 60, and then began gradually to drop, decreasing by approximately 50% on the 180th day, while the IgM titers progressively decreased as early as the tenth day, but they could be detected even at later time points. Despite the small number of individuals, some peculiar characteristics of the humoral response in COVID-19 emerged. We observed a high inter-individual variability, an ephemeral IgG half-life in several patients, and a persistence of IgM in others.

## 1. Introduction

In recent months, humanity is facing one of the most dramatic epidemics of the last few centuries, namely the one caused by the new coronavirus SARS-CoV-2. The development of effective prevention and treatment strategies for the corresponding disease, named COVID-19, cannot occur without an accurate understanding of the natural history of the disease.

SAR-CoV-2 is an enveloped, non-segmented, positive sense RNA virus, included in the *Coronaviridae* family. It is a novel β-coronavirus, after the previously identified SARS-CoV and MERS-CoV, with a diameter of about 65–125 nm, containing single strands of RNA and provided with crown-like spikes on the outer surface [1]. Structurally, SARS-CoV-2 has four main structural proteins including spike (S) glycoprotein, small envelope (E) glycoprotein, membrane (M) glycoprotein, and nucleocapsid (N) protein, and also several accessory proteins. S glycoprotein facilitates binding of envelope viruses to host cells by attraction with angiotensin-converting enzyme 2 (ACE2) expressed in lower respiratory tract cells [2].

SARS-CoV-2 is a respiratory virus that is spread mainly through close contact with ill subjects. The primary way of transmission is represented by breath droplets emitted by infected people. The disease can cause mild or more severe symptoms such as pneumonia, difficulty breathing, severe acute respiratory syndrome, kidney failure and even death [3].

According to Lippi et al. [4], five different phases can be distinguished during the progression of COVID-19. These phases are not clearly sequential, may affect only part of the patients (which is very variable depending on the geographical area and the type of epidemiological survey carried out), and include a number of non-exclusive features [5]. During the first phase, after the incubation period lasting between 2 and 11 days (6 days in average), the onset of disease may be characterized by influenza-like symptoms, from mild to moderate [6,7]. In this phase SARS-CoV-2 can replicate actively in the upper respiratory tissues with high infectivity. Some individual recover and some progress to the second phase. This one is characterized by progressive respiratory involvement with onset of pneumonia-like symptoms. The third phase is characterized by severe interstitial pneumonia with focal and systemic hyper-inflammation (cytokine storm), which may lead to acute respiratory distress syndrome (ARDS), and systemic inflammatory response syndrome (SIRS). During this phase, patients require medical treatment in sub-intensive care units. The fourth phase of COVID-19 develops in a relatively small number of patients, and is characterized by the onset of microvascular and macrovascular thrombosis possibly promoted by strong local and/or systemic inflammation. The last phase can evolve into two different outcomes: decease or remission.

SARS-CoV-2 infection outcome seems to be affected by a number of factors including environmental factors (climate, pollution, cultural, social and economic inequalities, climate, as well as health care system organizations), comorbidities (high blood pressure, cardiovascular disease, other heart and lung conditions, diabetes, cancer, or compromised immune systems), and inter-individual genetic differences [8,9,10,11,12].

The SARS-CoV-2 antigens stimulate the human immune system to produce IgM and IgG antiviral antibodies that are present in serum samples of patients [13]. Generally, IgM antibodies appear in the initial and acute phase of the disease, then progressively decrease and the IgG titer increases in the convalescence phase [14].

The significance of the detection of IgG and IgM through serological test depends on the antibody kinetics (seroconversion, decrease in IgM, appearance of IgG), on the persistence of antibodies over time, and on their immunogenicity. Current data confirm that the antibody kinetics of the SARS-CoV-2 is not still completely defined, and this applies to all phases of the disease. Results indicate that the antibodies (IgM and IgG) develop several days after infection (on average 10, and only 50% of patients present antibodies after 7 days) [15,16]. Furthermore, positivity to serological test might not be detectable in all patients.

On the other hand, antibody responses to other human coronaviruses were reported to wane over time [17]. Seroprevalence data deriving from the study of the SARS-CoV confirmed the positivity for specific IgG for a limited time (two years), which was followed (from the third year) by the negativization, leading to the possibility of reinfections after this period [18,19]. Multiple studies reported that while IgM and IgG titers increased during the first weeks following symptom onset, IgM levels gradually decreased for MERS-CoV in comparison to IgG levels about a month post follow-up. Other studies reported detectable IgG levels in recovered MERS-CoV patients at five months and one year after illness onset [20]. An endemic human coronavirus like HCoV-229E showed an increase in antibody level after 8 days, a peak around 14 days, and a wane after 11 weeks, although significant variation between patients was reported [21]. While, for HCoV-OC43 and HCoV-HKU1 a 45 weeks period of protective immunity was observed [17].

There is little information regarding the mechanisms and role of the humoral response in SARS-CoV-2 infection. Such information is obviously crucial for pandemic risk assessment, for designing effective monitoring strategies based on serological tests, for predicting vaccine efficacy, and for decision makers. In particular, a very important aspect is that of antibody kinetics and dynamics in symptomatic, pauci-symptomatic and asymptomatic COVID-19 patients. Defining this aspect would increase our knowledge of the natural history of the disease. In this study, a cohort of COVID-19 patients, confirmed by molecular test, were subjected to the detection of anti-SARS-CoV-2 IgM and IgG. The antibody levels were analyzed according to the clinical status (whether symptomatic, pauci-symptomatic, or asymptomatic), the time from the symptom onset, sex, age, and comorbidities.

## 2. Materials and Methods

### 2.1. Study Design and Participants

This study was part of the “COVID-19 Research Project” promoted by the Local Health Authority (ASL) of Lecce and the University of Salento. During the first COVID-19 pandemic, in the period between 28 February and 13 June 2020, 1793 subjects were screened for SARS-CoV-2 at the Clinical Pathology and Microbiology Unit of the “Vito Fazzi” Hospital in Lecce (Puglia, Italy). The examined subjects were heterogeneous, partly hospitalized in different clinical operating units (for other diseases), and partly medical and paramedical workers, which were subjected to health surveillance. The screening was performed by real-time RT-PCR for SARS-CoV-2 RNA through nasopharyngeal or oropharyngeal swab test and revealed 97 positive subjects. Subsequently, 54 (55.7%) of these, according to their consent and availability as well as the disposability of serological kits, underwent serological testing for anti-SARS-CoV-2 IgG and IgM detection from 9 April to 3 September 2020, constituting the studied cohort.

### 2.2. Data Collection

Demographics, symptom history, clinical characteristics and outcome data were obtained with standardized case report forms from the medical records of each subject. In particular, the form included data about: origin of the case (screening, contact tracing, etc.), personal data (date of birth, residence, etc.), epidemiological survey (place of exposure, related other cases, healthcare worker), laboratory analysis (molecular and serological tests), data of hospitalization, clinical features (onset of symptoms, comorbidities), clinical status (asymptomatic, pauci-symptomatic, mild, severe, critical).

### 2.3. Definitions

A confirmed case of COVID-19 was defined as an individual with nasopharyngeal swab that tested positive for SARS-CoV-2, using laboratory-based reverse transcriptase real-time PCR. The pauci-symptomatic patients were defined as patients with symptoms such as fever, cough, sore throat and fatigue; the patient who had mild symptoms were defined as patients with fever, respiratory symptoms and mild pneumonia; the patients with severe symptoms were defined as patients with difficulty breathing, hypoxia, abnormal blood gas analysis, and severe pneumonia; the critical patients were defined as patients with respiratory failure (severe acute respiratory syndrome) [22]. An asymptomatic case was defined as an individual with a positive nucleic acid test result but without any relevant clinical symptoms in the preceding 14 days.

### 2.4. Molecular Assays

Nasopharingeal swabs were tested by molecular assays to detect the presence of SARS-CoV-2 in the upper respiratory tract of subjects. Two methods were used: the one-step GeneFinder COVID-19 PLUS RealAmp Kit (OSANG Healthcare Co., Ltd., Anyangcheondong-ro, Dongan-gu, Anyang-si, Gyeonggi-do, Korea) and the Allplex 2019-nCoV Assay (Seegene, Seoul, South Korea). The former was used on the ELITe InGenius platform (ELITech, Torino, Italy) and integrated extraction and amplification. The RT-qPCR kit detected in the same PCR reaction the presence of three SARS-CoV-2 targets: the envelope protein (E), the nucleocapsid protein (N) and the RNA-dependent RNA polymerase (RdRp) genes. According to the producer instructions, a gene was considered detected when the cycle threshold (Ct) value resulted ≤43. The latter method detected the same genes and was performed on CFX-96 real-time thermal cycler (Bio-Rad Laboratories, Inc., Hercules, CA, USA). The fluorescence was measured using a Seegene Viewer (Seegene, Seoul, Korea).

### 2.5. Detection of Anti-SARS-CoV-2 IgG and IgM

The determination of IgG and IgM against SARS-CoV-2 S-antigen and N-protein was carried out in serum samples by chemiluminescence immunoassay using the MAGLUMI™ 2000 Plus 2019-nCoV IgM and IgG kit (Snibe, Shenzhen, China). The cutoff value for IgM positivity was 1.0 AU/mL, while that for IgG was 1.10 AU/mL.

### 2.6. Statistical Analysis

All data obtained from the case report forms were entered into a Microsoft Excel database and statistically analyzed by MedCalc Software version 12.3 (MedCalc Software bvba, Ostend, Belgium). A descriptive analysis was conducted: mean values and standard deviation (SD) were determined for continuous variables (age, IgM and IgG titers); frequency (%) was calculated for categorical variables. The association between some characteristics of recruited subjects (presence of comorbidities, age, sex) and the typology of COVID-19 symptoms (whether they were asymptomatic, pauci-symptomatic as well as with mild, severe, or critical symptoms) was evaluated by chi-square test. A multiple regression analysis was performed to determine the contribution of sex, age, clinical status, number of comorbidities, and time (days) from symptom onset to IgM and IgG concentration. A box plot showing the median, interquartile ranges, outliers and extremes was created with IgM and IgG values of each subject divided by clinical status. The comparison among the various categories were evaluated by the one-way ANOVA test. Differences were considered significant when *p* < 0.05.

A scatter graph (XY) was drawn to plot IgM and IgG titers from all subjects against days after symptom onset. IgM and IgG values were interpolated by polynomial approximations in order to highlight their trend over time and its confidence interval (95%).

### 2.7. Ethical Aspects

The study was approved by the Ethical Committee of the Lecce Local Health Authority (ASL/LE) on 29 May 2020 with deliberation n. 557. All data were collected and analyzed confidentially in accordance with Italian laws (Legislative Decree n. 196 of 30 June 2003, and subsequent additions), for research purposes.

## 3. Results

Characteristics of the analyzed COVID-19 patients are summarized in Table 1. Of the 1793 screened subjects, 97 (5.4%) resulted positive in the swab test. Fifty-four swab-positive patients (55.7%) underwent serological testing for anti-SARS-CoV-2 IgG and IgM detection once (38 patients, Table 2) or multiple times (16 patients, Table 3). This cohort was made up of 29 (53.7%) males and 25 (46.3) females. Forty (74.1%) subjects were patients hospitalized in different clinical operating units, while 14 (25.9%) were healthcare workers. The average age was 58 ± 18.4 years and 17 (31.5%) people were under 50 years. The youngest patient was 22 years old and the oldest was 95 years old. In relation to the symptoms that characterize COVID-19, 14 (25.9%) subjects of the cohort were asymptomatic, seven (13.0%) were pauci-symptomatic, 12 (22.2%) had mild symptoms, 12 (22.2%) had severe symptoms, and nine (16.7%) had critical symptoms. The mean age of asymptomatic patients differed from that of symptomatic patients (45.1 ± 15.9 years vs. 62.5 ± 17.2 years, *p* = 0.002). In addition, 27 (50.0%) subjects had some comorbidities (one to four simultaneously) (diabetes, cancer, cardiovascular diseases, chronic respiratory diseases, obesity, chronic neurological diseases, etc.). Regarding the severity of COVID-19, 9 subjects were admitted to intensive care, and three died.

Of the swab-positive subjects, 42 (77.8%) tested positive for the serological test (25 for IgG only, 2 for IgM only and 15 for both) and 12 (22.2%) tested negative. In the latter case, five (41.7%) subjects were asymptomatic, one (8.3%) was pauci-symptomatic, five (41.7%) had mild symptoms and only one had severe symptoms. The time interval between symptom onset and the serological testing ranged from a minimum of 25 days (corresponding to one of the patients with mild symptoms) to a maximum of 64 days (corresponding to the patient with severe symptoms) (Table 2).

Of the 42 subjects who tested positive for both swab and serological tests, nine (21.4%) were asymptomatic, six (14.3%) were pauci-symptomatic, seven (16.7%) had mild symptoms, 11 (26.2%) had severe symptoms and nine (19.6%) had critical symptoms. The time interval between the onset of symptoms and the first serological test in a given subject ranged from a minimum of 12 days to a maximum of 86 days.

Overall, among the asymptomatic subjects (*n* = 14) of the cohort, five tested negative and nine tested positive in the serological test.

In relation to the antibody titer, 10 patients had an IgG concentration greater than 20 AU/mL, 18 a concentration between 20 and 5 AU/mL, and 14 less than 5 AU/mL. The highest titer was 100.5 AU/mL in a 67-year-old male subject (#859) who underwent the serological test 59 days after the onset of symptoms. The lowest IgG titer was equal to 1.39 AU/mL, and was detected in a 52-year-old female subject who was asymptomatic (#1796) (Table 3). The minimum time that elapsed between the symptom onset and a positive serological test for IgG was 12 days and, in this case, the IgG titer was 4.50 AU/mL in a 62-year-old male subject (#2491), while the IgM titer was under the cutoff value (Table 2). After 86 days, which is the longer time interval between symptom onset and the positive serological test, the IgG titer was equal to 7.80 AU/mL in a 57-year-old male subject (#8) and, also in this case, the IgM was under cutoff value (Table 2). In Table 3, which reports data on patients who performed the serological test at least twice, there are four subjects (#572, #589, #667, #834) who showed positivity for IgG after more than 100 days from symptom onset, up to 169 days. Only one of them developed severe symptoms (#667).

Regarding IgM, 18 subjects had a concentration above the cutoff value (1.0 AU/mL). The highest titer was 7.00 AU/mL, which was found 59 days from symptom onset in the same subject who had the highest IgG titer (#859) (Table 2). The lowest IgM titer was 1.01 AU/mL, and it was detected in a 55-year-old male subject (#572) who performed the serological test 52 days after symptom onset (Table 3). Subject #2231 performed the serological test 13 days after symptom onset, and tested positive for IgM (5.65 AU/mL) but negative for IgG (0 AU/mL). This patient died 20 days after symptom onset and it was no possible to perform further tests. Moreover, nine subjects had persistent IgM, suggesting a possible role of IgM also during the advanced stages of the disease. Subjects #18, #753, #859 were IgM positive after 79, 36 and 59 days from symptom onset, respectively (Table 2); subjects #184, #199 and #572 resulted positive for IgM after 55, 56 and 52 days from symptom onset; subjects #589, #667 and #1107 showed positivity for IgM even after 125, 112 and 93 days from the onset of symptoms (Table 3). Five (55.5%) of these 9 patients who had persistent IgM developed severe symptoms, whereas the others had mainly mild symptoms.

The distribution of subjects with comorbidities and age ≥ 50 years appeared to be different (*p* < 0.05) within the various COVID-19 symptom groups (Table 4). On the contrary sex seemed to be not associated with clinical status. The prevalence of subjects with comorbidities as well as the subjects which had ≥50 years old increased with the severity of COVID-19 symptoms showing the maximum level among patients with critical symptoms (88.9% and 100.0% respectively).

**Figure 1:**
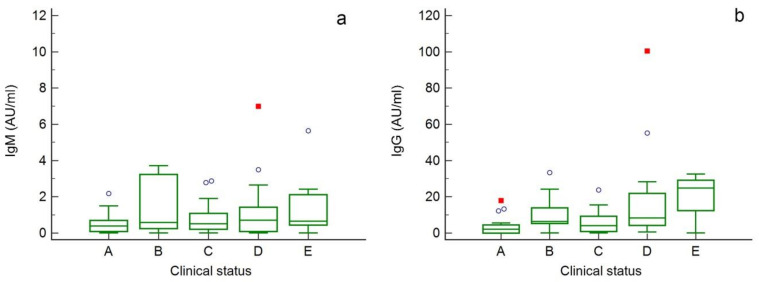
Box-and-whisker plots showing the distribution of IgM (**a**) and IgG (**b**) concentrations in the analyzed COVID-19 patients divided according to the severity of symptoms (A: asymptomatic; B: pauci-symptomatic; C: mild; D: severe; E: critical). Outliers were indicated in the figure as circles (outside values) and red squares (far out values).

Figure 1 shows the distribution of IgM (a) and IgG (b) concentrations in the analyzed subjects divided by the clinical status (A: asymptomatic; B: pauci-symptomatic; C: mild; D: severity; E: critical). Overlapping values for IgM among the different groups can be observed, with the median values ranging from 0.39 AU/mL in the asymptomatic subjects to 0.71 AU/mL in subjects with severe symptoms. Regarding IgG, instead, the distributions appear overall increasing according to the severity of the symptoms, with the medians ranging from 2.16 AU/mL in the asymptomatic patients to 24.87 AU/mL in subjects with critical symptoms.

Based on analysis of variance (one-way ANOVA) no significant difference in IgM level was found between groups (*p* > 0.05), whereas for IgG a significantly higher concentration (*p* < 0.05) was found in subjects who developed severe (18.5 ± 26.0 AU/mL) and critical (20.1 ± 11.4 AU/mL) symptoms compared to asymptomatic subjects (3.49 ± 4.94 AU/mL) (Table 5). However, it should be considered that patients with critical or severe clinical status underwent serological testing earlier than others (starting 12 days from symptoms onset vs. 25 days) and this might influence the level of antibodies detected.

**Figure 2:**
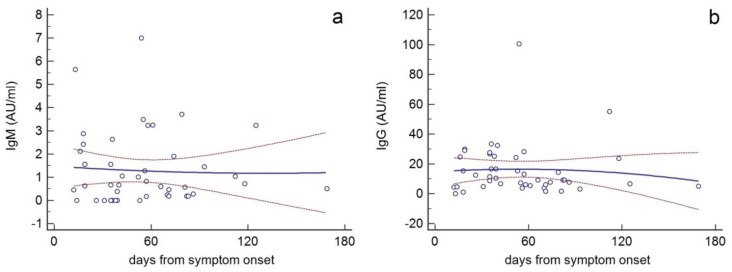
Scatter graphs (XY) whereby IgM (**a**) and IgG (**b**) titers (AU/mL) from all subjects were plotted against time (days after symptom onset). IgM and IgG values were interpolated by polynomial approximations (solid lines) with their confidence interval (95%) (red dotted lines).

In general, when IgG and IgM titers (AU/mL) from all subjects were plotted against time (days after symptom onset) (Figure 2), a second-degree polynomial approximation showed that overall IgG titers peaked around day 60, and then began gradually to drop, decreasing by approximately 50% on the 180th day (b). Polynomial interpolation also showed that IgM titers progressively decreased as early as the tenth day, but they could be detectable even on the 180th day (a).

Multiple regression analysis revealed that IgG concentration was significantly associated with clinical status (coefficient correlation = 4.029; standard error = 0.969; *p* < 0.01) and days from symptom onset (coefficient correlation = 0.0638; standard error = 0.0229; *p* < 0.01) and resulted independent from sex, age and number of comorbidities. Whereas IgM concentration was associated with none of the considered parameters.

However, antibody concentrations were highly variable, so it does not appear to be a clear relationship between antibody profile and time from symptom onset. In fact, with increasing days the concentration of IgG does not always increase, just as the concentration of IgM does not always decrease. This is also evident by examining the data of 16 patients who underwent serological testing multiple times at a distance of several days (Table 3).

In this subgroup, 12 subjects performed the serological test twice, two subjects for three times, two subjects for four times. The minimum interval between the symptom onset and the first serological test was 14 days, while the maximum was 74 days. The time interval between two tests ranged from 1 day to 102 days.

Eight subjects (#184, #199, #628, #834, #901, #1095, #1107, #1796) showed a decrease in the IgG titer after a very variable number of days (from 3 to 102 days); 4 patients (#1087, #667, #1781, #1563) showed an IgG titer increase after 32, 55, 58 and 62 days from the first serological test. The IgM titer decreased in 3 cases (#184, #1107, #572): after 7, 19 and 66 days from the previous test; while it increased in one case (#1781) after 58 days.

Patients #184 and #589 are of particular interest: the former showed a rapid decaying of both antibodies in the second test (7 days after the first), but in the third test (after 13 days) the IgG titer slightly increased and the IgM titer grew enormously; this subject underwent the serological test after 35 days from the onset of symptoms, which were severe, and recovered in 10 weeks from the symptom onset. The latter, also initially showed a slight decrease in IgG, but after 64 days (on the fourth test) showed an increase in IgG of 18%, while the IgM titer remained stable. This patient underwent serological test 2 days before the symptom onset, and, indeed, the first test was negative for both IgG and IgM. The first positive swab was recorded in the same day of the symptom onset. The second serological test resulted positive (after 58 days from symptom onset) and the IgG titer continued to increase even 125 days after the onset of symptoms.

Subject #667, after 55 days from the first positive serological test, showed almost a doubling of the IgG, and the IgM, which in the previous test were negative, became positive. There were 57 days between the onset of symptoms, that were severe, and the first serological test.

Subject #1107, after 19 days from the first serological test, showed a decline in IgG of 60% and in IgM of 25%. In this case, between the onset of (mild) symptoms and the execution of the first serological test there were 74 days.

A particular case is the subject #1342 who showed a negativization for IgM after only 7 days from the first positive test, while the IgG always remained negative. This subject is a healthcare worker who did not have symptoms; the swab resulted positive on the same day of the first serological test which was negative. Subsequent swabs, repeated a few days later, resulted always negative.

Noteworthy is subject #1618 who was positive for IgM on the first test; after 30 days (18 days after the symptom onset) the IgM titer remained stable but IgG also became positive. This subject performed the first serological test 12 days before the onset of symptoms and two days after the positive swab (not shown in the table). She had mild symptoms and recovered one month after diagnosis with molecular testing.

Finally, subject #1781 showed a 25% increase in IgM and a slight increase in IgG (8%) after 58 days from the first serological test. This subject was asymptomatic and tested positive for the swab 94 days before the first serological test was performed.

Among the patients of this subgroup, the highest antibody titer for IgG was 55.19 AU/mL (#667), while for IgM it was 3.49 AU/mL (#184).

The analysis of the present data shows that IgG and IgM resulted positive after 12 and 13 days from the onset of symptoms, respectively. Unfortunately, no earlier test was made. Overall, IgG and IgM were both positive up to 18 days from the onset of symptoms, and after this time point IgM resulted generally negative.

## 4. Discussion

In this study a cohort of 54 subjects positive to SARS-CoV-2 swab test was tested for the detection of serum antibody concentration. These individuals presented different symptoms, none to critical, and comorbidities (none more than four simultaneously).

Overall, the presence of comorbidities and the age of subjects were associated with their clinical status with an increased prevalence of subject with comorbidities and age ≥ 50 years among patients with more severe symptoms. This evidence was highlighted in other studies which stated that patients with COVID-19 disease who had comorbidities and older patients were more likely to develop a more severe course and progression of the disease with an increased admission rate into the intensive care unit (ICU) and mortality [23].

The framework provided by this study confirmed substantial inter-individual differences in the humoral immunity response to SARS-CoV-2, with a consequent difficulty in establishing a common profile to all cases of COVID-19 diseases for antibody kinetics and dynamics. Some of the inter-individual variations, also observed in the literature, could be resulted from representational biases owing to symptom-based case definition or lack of well-standardized analytical procedures or difference in methodologies [24]. However, the implication of genetic and non-genetic host factors behind these variations would seem important [8,9,10,11,12].

In the present study the date of the onset of symptoms of COVID-19 was considered as a reference time point to evaluate the serological profiling. The time interval between the symptom onset and the serological test in a given subject ranged from a minimum of 12 days to a maximum of 86 days, and the serological test (IgG and/or IgM) was positive in about 77.8% of patients. This value was consistent with that indicated in a previous study, in which positivity rate of IgM and IgG was reported to be about 85% and 78%, respectively, in the first week of symptom onset [25].

We observed that only about 64% of asymptomatic subjects tested positive in the serological test. Long et al. [26] suggested that asymptomatic individuals have a significantly weaker immune response to SARS-CoV-2 infection than symptomatic subjects.

Our data showed that IgG concentration was significantly associated with the clinical status and the time from symptom onset while resulted independent from sex and age of subjects as well as any comorbidities. In particular, the IgG level was higher in patients who developed critical or severe symptoms as demonstrated in another study, which underlined a strong positive correlation between clinical severity and antibody titer at 2 weeks after illness onset in patients with COVID-19 [27]. This finding suggests that a high antibody titer might be considered as a risk factor for critical illness.

Many studies addressed the problem of seroconversion of IgM and IgG in COVID-19 patients. It was reported that IgM seroconversion can be detected as early as 5 days following symptom onset, while IgG within 14 days [25]. However, a comprehensive study of the acute antibody response to SARS-CoV-2 in 285 patients with COVID-19 demonstrated that seroconversion of IgM and IgG in COVID-19 patients can occur simultaneously or sequentially, either preceded by IgM or even by IgG [28]. This study also confirmed that IgG titers in severe COVID-19 patients were significantly higher than those in non-severe patients within 8–14 days post-symptom onset, and showed that IgG titers decreased slightly during 15–21 days from the onset of symptoms in severe patients, while slightly increased in non-severe patients [28]. This means that some of the negative serological tests that were found in our screening could be attributed to either premature testing or drop of IgG titer below the cutoff value.

Indeed, a major point of interest is the strength and duration of humoral immunity in COVID-19 subjects. Long et al. [26] reported that IgG and neutralizing antibody levels start to decrease significantly within 2–3 months after infection. These data are also confirmed by Seow et al. [29], highlighting the transient nature of the antibody response towards SARS-CoV-2. This characteristic reflects more the immune response to endemic seasonal coronaviruses (i.e., those associated with the common cold) which were also reported to be transient. Another study demonstrated that in convalescent individuals (4 out of 8 examined subjects) neutralizing antibodies titers significantly dropped approximately 6–7 weeks after illness onset [30]. However, despite the waning of neutralizing antibody titers, it is possible that they will still be sufficient to provide protection from COVID-19 disease for a period [29]. Altogether these findings suggest a short duration of immunity following SARS-CoV-2 infection, as also predicted by mathematical model [31]. This may have important implications for widespread serological testing, re-infection to SARS-CoV-2, and the durability of vaccine protection.

In comparison, IgG levels peak at 4 months after the onset of SARS, 90% and 50% of SARS-CoV infected patients have been shown to maintain sustained IgG levels for 2 and 3 years, respectively [32]. Sustained IgG levels have been shown also in MERS-CoV infection with an antibody response lasting more than 34 months after the onset of symptoms [33].

The short-lived immunity approximates the humoral response to SARS-CoV-2 to that evoked by seasonal endemic coronaviruses known to induce a rapidly declining antibody response (within 12 and 52 weeks of disease initiation), and re-infection [34]. Here we show that when IgG and IgM titers were plotted against time (days after symptom onset), polynomial approximation showed that IgG titers peaked around day 60, and then began to gradually drop, decreasing by approximately 50% on the 180th day, while the IgM titers progressively decreased as early as the tenth day, but could be detected even at later time points.

However, considerable inter-individual variability was found as already observed [24]. Specifically, in the patient #184 decreases of IgG titer by 41%, and IgM titer by 33% after 7 days from the date of the first serological test were observed, with estimated titer half-lives of 9 days and 12.2 days for IgG and IgM, respectively. This short IgG half-life might be due to predominant IgG3 subclass production. In fact, it is well known that IgG3 subclass half-life is considerably shorter (about one third) than those of the other subclasses [35], and there is some evidence that, in contrast to other respiratory viral infections such as influenza, instead of IgG1, IgG3 appears to be the dominant subtype in SARS-CoV-2 infection [36]. Importantly, IgG3 is nephritogenic and avidly fixes complement, and its high molecular weight and anionic charge favor its localization in the glomerular capillary wall [37].

Another interesting aspect involves subjects #199, #2491, and #2675 who were expected to be positive for IgM, as they performed the serological test at 14, 12, and 19 days after symptom onset, respectively, when the antibodies are produced in sufficient amounts to be detected. Our results may suggest a possible failure of these patients to generate the antibody response, and this may have contributed to the disease severity, in fact they developed severe to critical conditions, and subject #2491 died.

On the other hand, the observed IgM persistent positivity in 9 out of the 17 IgM-positive subjects, after 35 up to 125 days from the symptom onset is also noteworthy. It is difficult to understand, in the absence of serological test repeated several times at different intervals of time on the same subjects, what is behind this phenomenon. However, we may hypothesize different scenarios: (i.) long-lasting IgM titers in these patients; (ii.) particularly strong primary IgM response; (iii.) re-infection; (iv.) infection persistence/re-activation. Several studies, in fact, describe cases of possible re-infection (or infection persistence/re-activation) by SARS-CoV-2 [38,39,40,41,42,43]. These cases generate concern, not only with regards to the ephemeral duration of the immune response in some subjects, but also because infection recurrence in recovered patients can favor the selection of escape mutants and their subsequent spread to the population [44].

IgM-mediated immunity is usually considered to be transient, prior to onset of high-affinity IgG, and therefore of little value for protection against re-infection. However, several studies have begun to challenge this notion demonstrating that IgM, produced either innately, or in response to antigen challenge, plays an important role not only in early immunity, but also in long-term protection against re-infection by a variety of pathogens [45]. The availability of a suitable animal model (genetically engineered mouse strains that produce only IgM, or isotype-switched IgG) has allowed to demonstrate that IgM production may be induced following re-infection, suggesting that the response is due to a memory cell response, not persistent antibody [45].

Moreover, it may be also relevant to note that some structural features make immune IgM particularly important in the antiviral response. It is well known that IgM is generated from germ-line configured information in B cells, prior to the onset of class switch recombination and somatic hypermutation, and is typically characterized by low affinity against the antigen. However high valency of secreted pentameric IgM can allow them to bind with a wide range of avidity, causing agglutination or clumping, a process that facilitates the removal of microbial pathogen or viruses. In fact, IgM are 100 to 10,000 times more effective than IgG in mediating agglutination, which are considered to play a key role in virus neutralization, especially if we consider that a single bound IgM molecule can activate complement and lyse erythrocytes, while thousands IgG molecules are usually required [46].

Indeed, natural and immune IgM, produced by B-1 and B-2 cells, respectively, are required for protection from influenza virus [47,48,49], early production of neutralizing immune IgM limits viremia and dissemination of West Nile virus [50], and natural IgM prevents dissemination and facilitates trafficking to spleen in Vesicular stomatitis virus infection by T cell-independent/complement-dependent neutralizing IgM response [51,52,53]. Altogether these findings suggest that more information on the role of the IgM-mediated response in SARS-CoV-2 infection would be highly desirable.

This study is not without limitations, such as the restricted number of patients analyzed and the lack of accurate serological monitoring over time (4 time points were carried out for only two patients). This may have produced an inter-individual variability (discussed above), since the time of appearance of antibodies may be affected by factors such as when the specimen was collected and when the symptom onset took place in each individual patient. Further studies using longitudinal sample collection in an unbiased manner to test the kinetics of the antibody response are required.

On the other hand, this study described the specific evolution of the infection in each subjects analyzed, which is less evident in a generalized description of the phenomenon, highlighting particular features of antibody response in COVID-19 patients, that could be used for future comparisons.

## 5. Conclusions

In this study, IgM and IgG antibody levels were analyzed in a cohort of 54 COVID-19 patients, some symptomatic, others pauci-symptomatic or asymptomatic. Overall, IgG concentration resulted associated with clinical status and time from symptom onset with increased values in subject with more severe symptoms. Despite the small number of enrolled subjects, some peculiar characteristics of the humoral response in COVID-19 patients, including high inter-individual variability, short IgG half-life in several subjects, and long-lasting IgM titers in others, emerged.

## Figures and Tables

**Table 1:** Descriptive characteristics of COVID-19 patients.

Variables	N (%)
Serological tests	54 (100.0)
Positive	42 (77.8)
Negative	12 (22.2)
Multiple serological tests	16 (29.6)
Sex	
Male	29 (53.7)
Female	25 (46.3)
Age	
<50 years	17 (31.5)
≥50 years	37 (68.5)
Clinical status	
Critical	9 (16.7)
Severe	12 (22.2)
Mild	12 (22.2)
Pauci-symptomatic	7 (13.0)
Asymptomatic	14 (25.9)
Subjects with comorbidities	27 (50.0)
Recovered	51 (94.4)
Died	3 (5.6)

**Table 2:** IgG and IgM titers in COVID-19 patients who underwent serological test only once.

Patient ID	Age (Years)	Sex	Days from Symptom Onset	IgG (AU/mL)	IgM (AU/mL)	Clinical Status
#550	65	M	40	32.41	NEG	Critical
#2145	66	F	19	29.89	1.56	Critical
#2231 *	66	F	13	NEG	5.65	Critical
#2333 *	69	M	16	24.66	2.12	Critical
#2491 *	62	M	12	4.50	NEG	Critical
#2675	78	M	19	29.17	NEG	Critical
#2776	85	M	26	12.48	NEG	Critical
#4915	58	M	18	15.36	2.42	Critical
#174	64	F	64	NEG	NEG	Severe
#753	74	M	36	16.62	2.64	Severe
#859	67	M	59	100.50	7.00	Severe
#888	83	F	81	1.75	NEG	Severe
#892	95	F	35	26.82	NEG	Severe
#1099	67	F	39	16.69	NEG	Severe
#2002	86	F	66	9.27	NEG	Severe
#2362	85	F	71	1.68	NEG	Severe
#2601	60	M	31	4.87	NEG	Severe
#8	57	M	86	7.80	NEG	Mild
#146	61	M	53	15.43	NEG	Mild
#226	53	M	70	3.79	NEG	Mild
#536	58	M	60	NEG	NEG	Mild
#1513	36	M	42	NEG	NEG	Mild
#1620	33	M	58	NEG	NEG	Mild
#4089	52	F	25	NEG	NEG	Mild
#4131	48	M	64	NEG	NEG	Mild
#18	56	M	79	14.39	3.71	Pauci-symptomatic
#676	32	F	57	13.23	NEG	Pauci-symptomatic
#685	76	F	36	33.39	NEG	Pauci-symptomatic
#894	89	F	35	8.78	NEG	Pauci-symptomatic
#2699	33	F	−2	NEG	NEG	Pauci-symptomatic
#25	56	F		12.32	NEG	Asymptomatic
#152	50	M		3.18	NEG	Asymptomatic
#338	31	M		NEG	NEG	Asymptomatic
#863	66	M		3.40	1.24	Asymptomatic
#1002	27	F		NEG	NEG	Asymptomatic
#1756	56	F		NEG	NEG	Asymptomatic
#3500	72	M		NEG	NEG	Asymptomatic
#4936	30	M		NEG	NEG	Asymptomatic

* died; M: male; F: female; NEG: negative.

**Table 3:** Trend of IgG and IgM in COVID-19 patients subjected to multiple serological tests.

ID Patient	Age	Sex	IgG (AU/mL)	IgM (AU/mL)	Days between Symptom Onset and Serological Test	Days between Serological Tests	Trend	Clinical Status
#901	73	M	27.7	NEG	35			Critical
			25.07	-	38	3	IgG ↓ 10%	
#184	78	M	11.58	1.56	35			Severe
			6.78	1.05	42	7	IgG ↓ 41%, IgM ↓ 33%	
			7.44	3.49	55	13	IgG ↑ 9%, IgM ↑ 232%	
#199	43	M	4.64	-	14			Severe
			3.88	1.27	56	42	IgG ↓ 16%	
#667	49	M	28.25	NEG	57			Severe
			55.19	1.04	112	55	IgG ↑ 95%, IgM +	
#572	55	M	24.23	1.02	52			Mild
			23.76	NEG	118	66	IgG =, IgM −	
#628	44	M	10.46	NEG	39			Mild
			9.29	NEG	82	43	IgG ↓ 12%	
			9.29	NEG	83	1	IgG =	
#1107	92	F	7.84	1.9	74			Mild
			3.18	1.45	93	19	IgG ↓ 60%, IgM ↓ 25%	
#1618	72	F	NEG	2.78	−12			Mild
			4.27	2.88	18	30	IgG +, IgM =	
#589	41	M	NEG	NEG	−2			Pauci-symptomatic
			6.07	3.23	58	60	IgG and IgM +	
			5.49	3.25	61	3	IgG ↓ 10%, IgM =	
			6.66	3.24	125	64	IgG ↑ 18%, IgM =	
#834	39	F	6.01	NEG	71			Pauci-symptomatic
			5.07	NEG	169	98	IgG ↓ 15%	
#1059	34	F	4.45	NEG	-			Asymptomatic
			3.29	NEG		20	IgG ↓ 25%	
#1087	47	F	13.33	-	-			Asymptomatic
			17.89	NEG		32	IgG ↑ 34%	
#1342	30	F	NEG	NEG	-			Asymptomatic
			NEG	2.18		3	IgM +	
			NEG	NEG		7	IgM −	
			NEG	NEG		68		
#1563	22	F	2.93	NEG	-			Asymptomatic
			5.56	NEG		62	IgG ↑ 47%	
#1781	58	F	4.10	1.11	-			Asymptomatic
			4.51	1.49		58	IgG ↑ 8%, IgM ↑ 25%	
#1796	52	F	1.39	NEG	-			Asymptomatic
			NEG	NEG		102	IgG −	

M: male; F: female; NEG: negative; ↓: decrease; ↑: increase; =: stable; +: become positive; −: become negative.

**Table 4:** Presence of comorbidities, sex and age of subjects within each COVID-19 symptom category.

COVID-19 Symptom Categories	Subjects with Comorbidities		Males		Subjects ≥ 50 Years Old	
	*n*	%	*n*	%	*n*	%
A	2	14.3	5	35.7	6	42.9
B	1	14.3	2	28.6	3	42.9
C	7	58.3	9	75.0	8	66.7
D	9	75.0	6	50.0	10	83.3
E	8	88.9	7	77.8	9	100.0
*p*-value *	0.0006		0.0933		0.0228	

* significance level determined by chi-square test; A: asymptomatic; B: pauci-symptomatic; C: mild; D: severe; E: critical.

**Table 5:** Mean IgM and IgG titer ± SD according to clinical status of COVID-19 patients and differences between groups.

Factor	Number of Tests	Mean IgM Titer ± SD (AU/mL)	Different (*p* < 0.05) from Factor *	Mean IgG Titer ± SD (AU/mL)	Different (*p* < 0.05) from Factor *
A	22	0.53 ± 0.55	-	3.49 ± 4.94	D, E
B	12	1.38 ± 1.49	-	10.3 ± 9.85	-
C	16	0.84 ± 0.92	-	6.13 ± 6.59	D
D	16	1.29 ± 1.81	-	18.5 ± 26.0	A, C
E	10	1.41 ± 1.70	-	20.1 ± 11.4	A

* significance level determined by one-way ANOVA test; A: asymptomatic; B: pauci-symptomatic; C: mild; D: severe; E: critical.

## Data Availability

The data presented in this study are available within the article (Table 2 and Table 3).
